# Case report: The efficacy of adding high doses of intravenous vitamin C to the combination therapy of atezolizumab and bevacizumab in unresectable HCC

**DOI:** 10.3389/fmed.2024.1461127

**Published:** 2024-10-03

**Authors:** Waleed Kian, Areen A. Remilah, Celine Shatat, Maria Spector, Laila C. Roisman, Larisa Ryvo

**Affiliations:** ^1^Institute of Oncology, Samson Assuta Ashdod University Hospital, Ashdod, Israel; ^2^Faculty of Health Sciences, Ben-Gurion University of the Negev, Beer Sheva, Israel; ^3^Helmsley Cancer Center, Shaare Zedek Medical Center, Jerusalem, Israel; ^4^Department of Radiology, Shaare Zedek Medical Center, Jerusalem, Israel

**Keywords:** vitamin C, ascorbic acid, hepatocellular carcinoma, liver cancer, atezolizumab, bevacizumab

## Abstract

**Introduction:**

Vitamin C (L-ascorbic acid) plays a vital role in human physiology, serving as both an antioxidant and a cofactor in enzymatic reactions. High-dose intravenous Vitamin C can achieve significantly elevated plasma concentrations, potentially enhancing its anticancer effects. This case study explores the synergistic impact of high-dose intravenous vitamin C in combination with bevacizumab and atezolizumab in the treatment of a patient with unresectable hepatocellular carcinoma (HCC).

**Case presentation:**

A 68-year-old male was diagnosed with unresectable HCC, presenting with elevated liver enzymes and an alpha-fetoprotein (AFP) level of 2018 ng/mL. Initial treatment with atezolizumab and Bevacizumab commenced in February 2022. Although imaging indicated stable disease, AFP levels decreased modestly to 1,526 ng/mL, while liver function tests remained elevated, accompanied by further clinical deterioration and weight loss. Subsequently, intravenous vitamin C (30 grams) was introduced into the treatment regimen. This addition led to a rapid and significant reduction in AFP levels, normalization of liver function tests, and marked improvement in clinical symptoms. The patient continued on this combined regimen of vitamin c, atezolizumab, and bevacizumab. Four months later, CT scans revealed significant tumor shrinkage and necrosis. As of 30 months post-diagnosis, the patient remains on the regimen with normal liver function and an AFP level of 1.8 ng/mL, maintaining normal activities and stable weight.

**Conclusion:**

To our knowledge, this is the first reported case of combining high-dose intravenous vitamin C with Bevacizumab and atezolizumab, which proved to be safe and resulted in significant clinical and radiological improvements in unresectable hepatocellular carcinoma (HCC). Further studies are recommended to explore the potential of this combination therapy.

## Introduction

Vitamin C, also known as L-ascorbic acid, is a crucial supplement. It is composed of six asymmetrical carbon atoms (C6H8O6) and is structurally related to glucose (1). Vitamin C is abundant in fruits and vegetables. Unlike many animals that can synthesize their own vitamin C, humans, guinea pigs, and fruit bats are unable to do so. This is due to a mutation in the L-gulonolactone oxidase (GULO) gene, which codes for the enzymes responsible for the final step of its synthesis (2, 3).

Vitamin C is a hydrophilic molecule that plays an essential role in human physiology (1). It acts as an electron donor and reduces the levels of dangerous reactive oxygen species (ROS) that are naturally produced during cell metabolism. This helps prevent certain diseases such as cancer and cardiovascular diseases (4).

In addition to its antioxidant function, vitamin C is an essential co-factor in various enzymatic reactions that contribute to collagen synthesis (5). A deficiency in vitamin C can lead to a disease called scurvy, which is characterized by widespread connective tissue weakness and capillary fragility. However, vitamin C also promotes ferritin production by improving the absorption of iron in the intestines (4, 5).

Beyond its physiological functions, ascorbic acid has been investigated for its potential role in preventing and treating cancer. Fifty years ago, Cameron and Pauling published numerous studies demonstrating the clinical efficacy of high-dose ascorbic acid in tumor regression and growth inhibition, leading to extended survival periods (6). In these studies, terminal cancer patients were administered a high dose of 10 g of ascorbic acid intravenously for 10 days, followed by an equal dose given orally (7, 8). Additionally, in palliative care, high-dose intravenous vitamin C (IVC) may be considered a therapy that improves quality of life and alleviates cancer-related symptoms such as fatigue and bone pain (9). It is important to note that subsequent studies on oral administration have not yielded precise and encouraging conclusions (10). Recent research has actually shown that the method of administration significantly affects the pharmacokinetics of ascorbate and its anti-cancer properties. Specifically, intravenous infusion bypasses the regulation of intestinal absorption that occurs with the oral route, resulting in high and uncontrolled plasma concentrations. However, rapid renal clearance leads to a half-life of approximately 2 h (9). Based on these findings, high dose IVC has emerged as a potential treatment for cancer, either on its own or in combination with conventional therapies. Additionally, multiple studies have demonstrated its tolerability and safety profile in pain management (11).

Several mechanisms of ascorbic acid’s anti-tumor activity have been reported. It acts as a modulator of the tumor micro-environment by regulating immune cell activity and epigenetic programs. Additionally, it has a pro-oxidant effect, generating reactive oxygen species (ROS) that contribute to cell apoptosis (12).

High-dose Vitamin C reduces iron from Fe3+ to Fe2+ through electron donation. The resulting Fe2+ reacts with hydrogen peroxide (H2O2), leading to the formation of hydroxyl radicals (OH•). These radicals contribute to cell death through the Fenton reaction. Additionally, Vitamin C enhances and restores the activities of TET enzymes, which trigger DNA demethylation and allow the re-expression of suppressor genes in tumor cells (13).

Furthermore, data have shown that the levels of ascorbate (Vitamin C) have an inverse correlation with HIF-1-alpha, which is responsible for cellular adaptation to hypoxia. Similar to TET enzymes, HIF hydroxylases require Vitamin C as a cofactor to regenerate Fe2+, thus inhibiting tumor growth (12, 13).

The synergistic anti-cancer activity of Vitamin C is a heavily researched area, supported by positive data collected from animal and preclinical trials. This research has progressed to phase 1 and 2 trials involving different types of cancers. Administering Vitamin C concurrently with various chemotherapeutic agents and radiation therapy has shown improvements in disease control and response rates (11). For instance, Ma et al. reported a median time of disease progression that was 8 months longer when combining ascorbate with standard chemotherapy in stage III/IV ovarian cancer. However, despite these findings, Vitamin C has not yet established itself as a standard of care (14).

Another successful aspect of cancer therapy is immune checkpoint therapy, specifically CTLA-4 and PD/PDL1 inhibitors. Emerging evidence suggests that Vitamin C can enhance the effects of immune checkpoint therapy by increasing the presence of macrophages and CD8 T-cells in the tumor microenvironment, leading to pronounced regression (11, 15).

Here, we report a case of combining an anti-VGFR (bevacizumab) and immune checkpoint inhibitor (atezolizumab) with high vitamin C in a patient with unresectable hepatocellular carcinoma (HCC).

### Case presentation

A 68-year-old male patient presented to our center with symptoms of loss of appetite, abdominal pain, and weight loss. There was no history of alcohol consumption or drug abuse. His medical history included hypertension, dyslipidemia, and ischemic heart disease. The family history was negative for any malignancy.

During the abdominal examination, a firm mass was found in the epigastric region. The abdomen was non-tender and non-distended, with no splenomegaly, ascites, or jaundice detected. Blood tests revealed mild anemia, with a hemoglobin level of 12.5 g/dL and a platelet count of 275,000/uL. Other laboratory findings included normal total serum bilirubin (0.7 mg/dL), alanine transaminase (22 IU/L), elevated aspartate transaminase (92 IU/L), GGT (1,043 IU/L), and alkaline phosphatase (560 IU/L). The albumin level was 3.8 g/dL, and the INR was slightly elevated at 1.19. Hepatitis B & C viral serology, as well as HIV tests, were negative. Serum tumor markers included alpha-fetoprotein (AFP) at 2018 ng/mL, CEA at 1.5 ng/mL, and CA19-9 at 22.8 u/mL.

**Figure 1:**
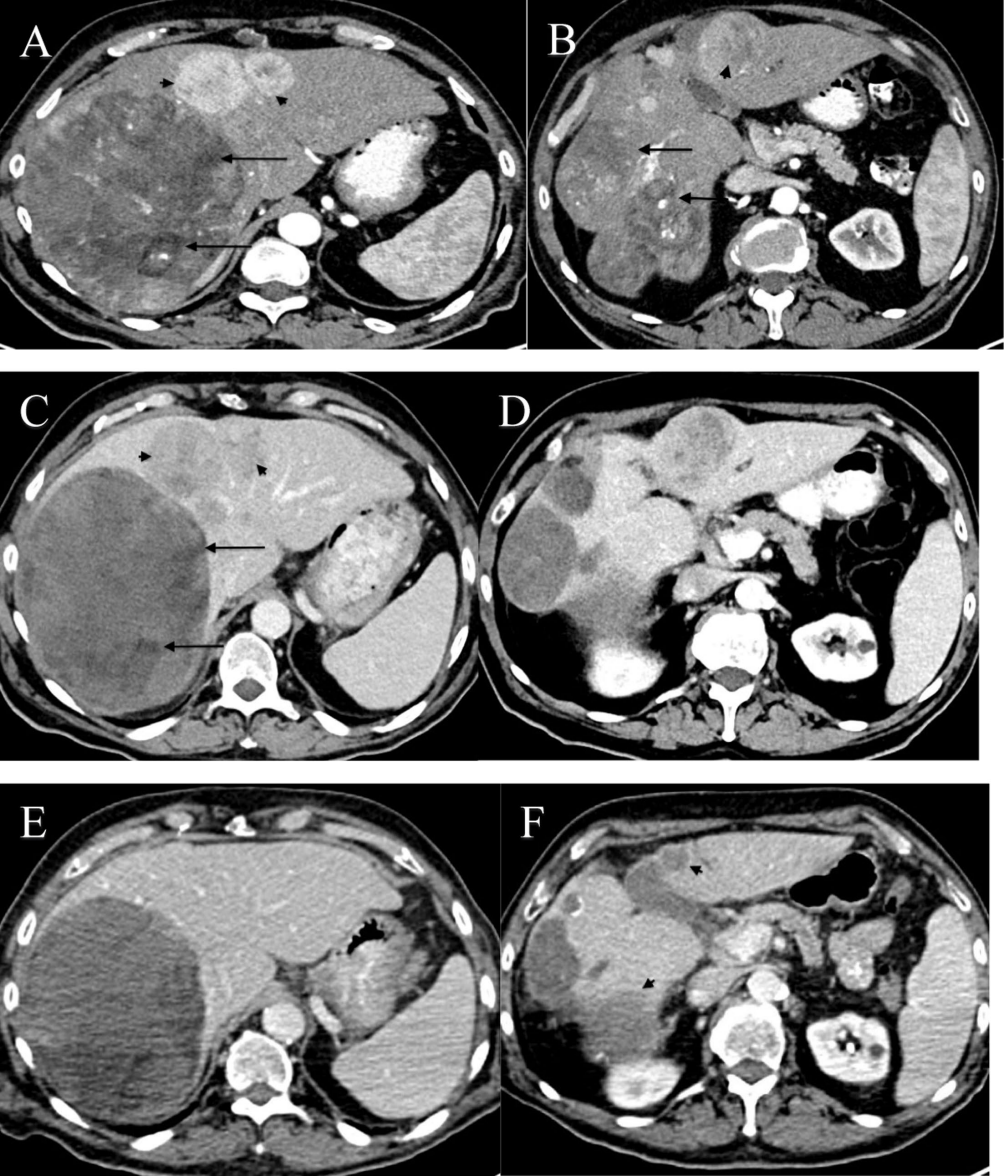
**(A–F)** Axial CT images of the abdomen at two levels through the liver showing multiple intraparenchymal masses with imaging features consistent with HCC, obtained during different stages of treatment. **(A,B)** Axial CT images at two levels before initiation of treatment, during the early arterial phase. **(A)** CT scan demonstrates a large anterior enhancing lesion in the right lobe of the liver with a mosaic pattern and low-density areas consistent with necrosis and fatty material (black arrows). Additional smaller arterial enhancing lesions were noted in the left lobe (black arrowheads). The liver did not demonstrate a cirrhotic pattern, prompting a biopsy. No portal venous involvement was noted. **(B)** A lower image through the liver shows lobulated heterogeneous masses (black arrow) in segments VI/VII and a well-defined mass with strong enhancement (arrowhead). **(C,D)** Axial CT images at the same levels, three months after initiation of treatment with atezolizumab and bevacizumab, obtained during the late arterial phase. Images demonstrate low attenuation areas in the peripheral portion of segment IV (black arrows) and two heterogeneous enhancement masses with no significant difference in size (black arrowheads). **(E,F)** Axial CT images at the same levels, two years after initiation of treatment with IV Vitamin C, obtained during the porto-venous phase. Images show an interval decrease in size (black arrowheads) and resolution of some lesions, suggestive of a complete response.

Contrast-enhanced abdominal tomography revealed a large, heterogeneous enhancing mass measuring 14 × 14 cm in the right lobe of the liver. Multiple additional lesions, measuring up to 4 cm, were observed in both lobes with arterial enhancement or venous washout. No extrahepatic lesions were seen (Figures 1A,B).

A liver biopsy revealed hepatic tissue with a trabecular pattern, displaying cytologic atypia and scattered mitosis. Immunohistochemical (IHC) stains were positive for Glypican 3, HSP, and Glutamine synthetase, strongly suggesting hepatocellular carcinoma with a Child-Pugh score of A. A gastroscopy was performed, which ruled out esophageal varices. This case was discussed at a multidisciplinary tumor board, and systemic therapy with Atezolizumab 1,200 mg and Bevacizumab 15 mg/kg was initiated in February 2022.

Subsequent 4-monthly surveillance showed a drop in AFP levels to 1,526 ng/mL. A CT scan in May 2022 revealed no change in the liver lesions (Figures 1C,D). However, hepatic function tests remained elevated, with a normal total bilirubin level. Clinically, the patient experienced gradually increasing fatigue and weakness, along with significant loss of appetite and weight loss of 12 kilograms.

**Figure 2:**
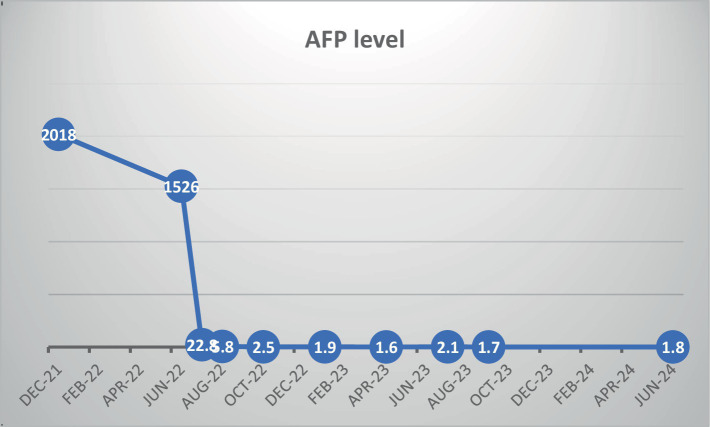
Alfa-fetoprotein levels throughout the treatment.

Due to the lack of radiological response (stable disease) and the patient’s gradual clinical deterioration, we recommended adding palliative intravenous vitamin C (30 grams) to the treatment regimen. Vitamin C was administered every 3 weeks in conjunction with atezolizumab and Bevacizumab. Before introducing vitamin C, baseline blood analyses, including liver function tests and AFP level measurements, were performed. Three weeks after the addition of vitamin C, a significant decrease in AFP levels and normalization of liver function tests were observed, as shown in Figure 2. Clinically, the patient reported a marked increase in energy, a notable improvement in appetite, and weight gain, with no noticeable adverse effects. Due to the significant clinical benefits and decreased AFP levels, vitamin C continued to be added to the atezolizumab and Bevacizumab protocol. A CT scan performed 4 months later revealed remarkable radiological shrinkage of all liver tumor lesions, with necrosis observed in the largest lesion. Blood tests showed normal liver function tests and an AFP level of 2.5 ng/mL.

In February 2023, the patient underwent a PET-CT scan, which revealed further shrinkage of liver masses. However, there was elevated FDG uptake in the largest mass, which could indicate necrosis or a viable disease remnant. The patient’s case was discussed by a hepatic multidisciplinary team, including a hepatologist, hepatobiliary surgeon, diagnostic radiologist, interventional angiographer, radiotherapist, and oncologist. Three treatment options were considered: observation, interventional thermo-ablation therapy, or liver Stereotactic Body Radiation Therapy (SBRT). After discussing these options with the patient, SBRT treatment was chosen. In April 2023, the patient received three fractions of 16 Gy each, for a total of 48 Gy.

Currently, 30 months after diagnosis, the patient continues to receive intravenous vitamin C every 3 weeks, along with atezolizumab and bevacizumab. The PET-CT scan shows no evidence of abnormal FDG uptake or new lesions (Figures 1E,F). Additionally, the patient’s liver function tests are normal, with an AFP level of 1.8 ng/mL. Clinically, the patient has returned to normal daily activity and has maintained his body weight.

## Discussion

Hepatocellular carcinoma (HCC) is a highly aggressive and fast-growing cancer, often resulting from chronic liver disease. While hepatitis B and C remain the primary causes of HCC globally, non-viral causes are becoming increasingly significant, especially in developed countries. Non-alcoholic fatty liver disease (NAFLD), driven by metabolic syndromes such as obesity and diabetes, is emerging as a major risk factor, with its incidence expected to rise dramatically in the coming years. Additionally, alcohol-related liver disease, genetic predispositions like hemochromatosis, and environmental toxins (such as aflatoxins) contribute to HCC development. These non-viral etiologies highlight the need for diverse preventive strategies (16).

Immunotherapy has made significant advancements in recent years, offering new treatment options for HCC (17). The rapid progress in synthetic biology and genetic engineering has facilitated the development of novel immunotherapies, including anti-programmed cell death-1 (PD-1) antibodies, anti-cytotoxic T lymphocyte antigen-4 antibodies, and anti-PD ligand 1 cell death antibodies. These treatments are now utilized for the treatment of advanced HCC (18).

The IMbrave150 trial demonstrated that the combination of atezolizumab (anti-PD-L1) and bevacizumab (anti-vascular endothelial growth factor (VGFR)) provided a clinically significant treatment benefit compared to sorafenib. This was evident in the improved overall survival rate, with a median of 19.2 months compared to 13.4 months. As a result, this combination has become the standard of care for the first-line treatment of metastatic HCC (19).

Recently, the HIMALAYA trial showed that a single dose of tremelimumab (anti-CTLA-4) combined with durvalumab (anti-PD-L1) significantly improved overall survival compared to sorafenib. The median overall survival was 16.56 months for the combination therapy, compared to 13.77 months for sorafenib (20). Consequently, on October 21, 2022, the FDA approved the combination therapy for the first-line treatment of unresectable hepatocellular carcinoma (21).

A post-hoc analysis of the IMbrave150 trial indicates that the biology of hepatocellular carcinoma (HCC) may be influenced by various etiologies, such as viral infections, alcohol-related liver disease, and metabolic conditions like nonalcoholic fatty liver disease (NAFLD). Contrary to prior conclusions from preclinical models and retrospective studies suggesting that NAFLD-related HCC might resist immune checkpoint inhibitors (ICIs), this patient-level analysis found no significant differences in overall response rate (ORR), progression-free survival (PFS), or overall survival (OS) across liver disease etiologies, challenging earlier assumptions about NAFLD’s impact on ICI outcomes (22–25).

Despite previous debates, there is a growing understanding that vitamin C possesses anticancer properties, particularly when administered intravenously and at high doses. Magri et al. discovered that the effects of vitamin C were significantly enhanced in the presence of a functioning immune system, and it synergized with checkpoint immunotherapy in immunocompetent mouse models of cancer (21). These findings suggest a promising avenue for combination treatment, which now needs evaluation in patients.

We present the case of a patient with a large, multifocal, unresectable HCC associated with NAFLD. The patient received a combination of bevacizumab and atezolizumab, achieving radiological stable disease and a modest decrease in alpha-fetoprotein (AFP) levels after four months. Due to minimal response, intravenous high-dose vitamin C (30 grams) was introduced as a palliative measure, which resulted in a marked decrease in AFP levels and normalization of liver function within three weeks. Given these positive outcomes, vitamin C was continued alongside the immunotherapy. Currently, two years post-diagnosis, the patient exhibits no signs of disease on PET-CT, with normal liver function tests and an AFP level of 1.7, as illustrated in Figure 2. The patient has resumed normal daily activities and maintained stable body weight without any signs of disease progression.

This case is notable as it appears to be the first reported instance of combining an anti-VEGFR agent (bevacizumab) and an immune checkpoint inhibitor (atezolizumab) with high-dose vitamin C in an unresectable HCC patient. The patient exhibited a rapid and sustained response, showing no evidence of disease two years after the initial diagnosis. This highlights the potential for vitamin C to complement immunotherapy in NAFLD-related HCC and underscores the need for further investigation into this combination therapy in similar patient populations.

## Conclusion

The combination therapy of high dose vitamin C with atezolizumab and bevacizumab has shown to be safe and efficacious. This may be attributed to the immune cell enhancement provided by vitamin C. However, further and larger studies are required to thoroughly evaluate and confirm the safety and efficacy of high dose vitamin C in conjunction with immunotherapy. These studies will help provide a more comprehensive understanding of the potential benefits and risks associated with this treatment approach.

## Data Availability

The datasets presented in this study can be found in online repositories. The names of the repository/repositories and accession number(s) can be found in the article/supplementary material.
